# Why is it so hard to implement change? A qualitative examination of barriers and facilitators to distribution of naloxone for overdose prevention in a safety net environment

**DOI:** 10.1186/s13104-016-2268-z

**Published:** 2016-10-18

**Authors:** Mari-Lynn Drainoni, Elisa A. Koppelman, James A. Feldman, Alexander Y. Walley, Patricia M. Mitchell, Jacqueline Ellison, Edward Bernstein

**Affiliations:** 1 Boston University School of Public Health, 715 Albany Street, T3 W, Boston, MA 02118 USA; 2 Boston University School of Medicine, Boston, MA USA; 3 Center for Healthcare Organization and Implementation Research, ENRM Veterans Administration Hospital, Bedford, MA USA; 4 Boston Medical Center, Boston, MA USA

**Keywords:** Opioid overdose, Overdose prevention, Policy implementation, Emergency department

## Abstract

**Background:**

The increase in opioid overdose deaths has become a national public health crisis. Naloxone is an important tool in opioid overdose prevention. Distribution of nasal naloxone has been found to be a feasible, and effective intervention in community settings and may have potential high applicability in the emergency department, which is often the initial point of care for persons at high risk of overdose. One safety net hospital introduced an innovative policy to offer take-home nasal naloxone via a standing order to ensure distribution to patients at risk for overdose. The aims of this study were to examine acceptance and uptake of the policy and assess facilitators and barriers to implementation.

**Methods:**

After obtaining pre-post data on naloxone distribution, we conducted a qualitative study. The PARiHS framework steered development of the qualitative guide. We used theoretical sampling in order to include the range of types of emergency department staff (50 total). The constant comparative method was initially used to code the transcripts and identify themes; the themes that emerged from the coding were then mapped back to the evidence, context and facilitation constructs of the PARiHS framework.

**Results:**

Acceptance of the policy was good but uptake was low. Primary themes related to facilitators included: real-world driven intervention with philosophical, clinician and leadership support; basic education and training efforts; availability of resources; and ability to leave the ED with the naloxone kit in hand. Barriers fell into five general categories: protocol and policy; workflow and logistical; patient-related; staff roles and responsibilities; and education and training.

**Conclusions:**

The actual implementation of a new innovation in healthcare delivery is largely driven by factors beyond acceptance. Despite support and resources, implementation was challenging, with low uptake. While the potential of this innovation is unknown, understanding the experience is important to improve uptake in this setting and offer possible solutions for other facilities to address the opioid overdose crisis. Use of the PARiHS framework allowed us to recognize and understand key evidence, contextual and facilitation barriers to the successful implementation of the policy and to identify areas for improvement.

**Electronic supplementary material:**

The online version of this article (doi:10.1186/s13104-016-2268-z) contains supplementary material, which is available to authorized users.

## Background

The increase in opioid overdose deaths in the United States has led to recognition of opioid overdose as a major public policy and public health issue. Opioid use in the US is at unprecedented levels and opioid overdose is epidemic. Rates of death from drug overdoses have more than doubled in the past 20 years [1], surpassing motor vehicle crashes as the leading cause of accidental death among persons aged 25–64, with 43,982 deaths in 2013 [2–4]. Opioids are the most common drugs involved in overdose deaths, and include both deaths from opioid analgesics and from heroin.

Massachusetts has seen a sharp increase in opioid overdoses and opioid overdose fatalities in recent years. In 2014, the state experienced a record number of deaths from unintentional overdose at 1256, a 57 % increase from 2012, with the overdose rate exceeding the national rate in 2013 and 2014 [5]. Declaring opioid overdose a public health emergency, the state has called for an urgent public health response and a broad and multi-pronged approach to addressing the rising and record high number of opioid overdose fatalities in the state [6]. It has become clear that new and innovative strategies are needed.

Access to and use of naloxone–commonly referred to by its brand name, Narcan™–is an important tool in opioid overdose prevention. Naloxone, an opioid antagonist, can immediately reverse an overdose upon administration. Distribution of Naloxone has been found to be a safe, feasible, and effective intervention in community settings, primarily in harm reduction and substance abuse treatment programs [7]. Laypersons, following a brief training, can safely administer intranasal naloxone to reverse overdose [8, 9]. Access to naloxone for community members has been further promoted, in Massachusetts and many other states, by the establishment of “standing orders” which are used in various situations to permit dispensing of naloxone rescue kits to laypeople without direct interaction with the prescriber [10, 11]. A pharmacy standing order is an agreement between a pharmacy and prescriber that permits the pharmacy to dispense naloxone rescue kits to people without a prescription or a direct relationship between the prescriber and pharmacy customer based on criteria agreed upon by the prescriber and pharmacy [12]. A hospital standing order is similarly an agreement between an inpatient pharmacy and a prescriber to dispense a naloxone rescue kit to patients upon discharge who meet pre-specified criteria, but do not have a direct relationship with the signatory of the standing order.

The demonstrated efficacy of order sets, pre-specified lists of treatment orders for a specific diagnosis, [13] provides insight into the potential value of standing orders for naloxone kit provision. Effective integration of order sets in hospital settings has been shown to improve clinical processes and patient outcomes for a range of medical conditions, [14, 15] though little is known about the utility of standardized processes for dispensing naloxone in these settings. Literature on order sets emphasizes the importance of provider determinants of successful integration including clinician satisfaction and perceived value [16]. A recent survey found that, despite barriers, emergency medicine physicians were willing to perform opioid harm reduction interventions [17]. Given the essential role of other clinical staff in care provision, successful integration efforts must take into account pharmacist, nurse, and other clinician’s perceptions of naloxone distribution via standing order.

High and increasing levels of opioid overdose present to emergency departments (EDs): in the US in 2011, there were 420,040 ED visits related to narcotic pain medications, an increase of 153 % from 2004. In addition, 258,482 ED visits were related to heroin, up from 187,493 in 2005 [18, 19]. The ED, often the initial point of contact for care for opioid-related issues, is a potentially high-yield location for delivery of overdose prevention activities.

As one potential measure to save lives from opioid overdose, one Boston safety net hospital introduced an innovative policy for nasal naloxone distribution. The ED partnered with local and state public health agencies to ensure that every patient seen at the ED with or at risk for opioid overdose was offered overdose education, training on the use of nasal naloxone and take home naloxone. Offering overdose prevention education and naloxone rescue kits to people likely to be present at an overdose is consistent with APHA, CDC, SAMHSA and Office of National Drug Control Policy recommendations [20, 21]. In this study, we used the Promoting Action on Research Implementation in Health Services (PARiHS) framework to examine the facilitators and barriers to the policy’s implementation.

## Methods

### Study context

We had two aims in this study: (1) understand the acceptance and uptake of the policy; and (2) identify facilitators and barriers of success. To address these aims, we obtained data on nasal naloxone distribution in the ED before and during the initial year of policy implementation, and then conducted a qualitative study to identify the facilitators and barriers to uptake. At the time of implementing the new initiative, the ED treated approximately 30 patients for overdose per month, in addition to a large number of patients with opioid use disorder diagnoses. A new policy to address overdose policy was promulgated in September 2013, with implementation beginning in January 2014.

Prior to the new policy, the model of care for overdose education and naloxone distribution was referral of opioid-using patients to a dedicated ED program that used Health Promotion Advocates (HPAs) available from 9 a.m. to 11 p.m. daily whose primary responsibility was referral to detoxification or substance abuse treatment, which has been previously described [22]. In the 8 months prior to policy implementation (January –September 2013) approximately 8 % of ED patients considered at risk for overdose (based on ICD-9 ED discharge codes associated with opioid overdose or opioid misuse/dependence; see Additional file 1 for a list of codes used to establish the denominator) received naloxone rescue kits in the ED. The low distribution rate was attributed to staffing patterns, patient factors, procedural issues, and lack of knowledge and/or support for the intervention. Institution of the new policy meant that naloxone kits could be distributed upon discharge from the ED during times when the HPAs were not on duty (11 p.m.–9 a.m.) and when outpatient pharmacies were closed. Naloxone kits were therefore available 24 h per day via one of three methods: (1) the HPA program; (2) outpatient pharmacy prescriptions written in the ED; or (3) inpatient pharmacy distribution by ED staff via the hospital standing order during times when the HPAs were not available or the outpatient pharmacy was closed.

At the time of policy development, overdose risk was broadly defined, and although there were guidelines in the policy (see Additional file 2 for a copy of the policy), interpretation was left to the ED provider’s discretion. The goal of the new policy was broader distribution of naloxone. Yet, data from the initial 8 months post-initiation of the policy (October 2013–May 2014) showed no expansion, with 7 % of emergency department patients with the same overdose risks based on the ICD-9 codes described above receiving take home naloxone in the ED. Thus, it was important to understand the barriers to policy adoption and what might facilitate better uptake of the policy x.

### Study design and conceptual framework

We conducted formative evaluation to understand barriers and facilitators to policy adoption. We used qualitative methods to assess the perspectives of a wide range of participants involved in the provision of ED care. To design our data collection instrument, we used the original Promoting Action on Research Implementation in Health Services (PARiHS) framework [23] which views implementation success as a function of relationships among three broad interrelated elements: evidence, context, and facilitation. According to PARiHS, the nature and communication of ***evidence***, qualities of ***context***, and appropriateness/technical aptitude of ***facilitation*** all determine how implementation outcomes are achieved. Most successful implementation occurs when there is a high level of each of the three elements. The construct of ***evidence*** goes beyond research evidence to include clinical experience, patient experience and local data or information. ***Context*** includes culture, leadership and evaluation. ***Facilitation*** relates to the process of enabling the implementation and includes the role played by the leader, as well as the skills and attributes possessed by that leader.

The semi-structured interview guide addressed issues related to the elements of evidence, context and facilitation. We focused on building our understanding of what individual interview and focus group participants believed about naloxone and its utility for overdose prevention, and beliefs about drug use and overdose. Further, we explored contextual factors of the ED that impacted policy implementation, including staff roles and responsibilities, logistical challenges, patient challenges, and the processes by which the facilitation of the policy occurred. Some specific areas (see Additional file 3 for guide) targeted during the interviews included knowledge of the policy, feelings about feasibility, target populations, provider roles and responsibilities, experience with naloxone, knowledge and use of the HPA program, and training.

### Data collection procedures

The ED employs a broad range of staff; our goal was to obtain a sample that included the perspectives of that range of staff members. Due to scheduling and roles, we conducted both focus groups (if at least three individuals in the same target population could attend) or individual interviews where more appropriate, i.e., physician administrators, nurse managers. In total, we conducted seven focus groups and six individual qualitative interviews using the same interview guide. Interviews and focus groups were conducted with ED leadership, attending and resident MDs, nurse management, nurse educators, staff nurses, HPAs and pharmacists. Data collection was conducted in private conference spaces between June and August 2014. Individual interviews lasted between 24 and 36 min with a mean and median of 30 min; focus groups lasted between 24 and 43 min with a mean of 31 min and a median of 30 min. All interviews and focus groups were conducted by two members of the study team and were audio-taped. Each participant also completed a brief anonymous demographic information form. Verbal informed consent was obtained from all study participants, as approved by the Boston University Medical Center IRB.

### Data analysis

After each interview was completed, the audio-tape was sent to a professional transcription company, and transcribed verbatim for analysis. Following receipt of the transcribed interview, the researchers reviewed the transcripts for accuracy of transcription. To conduct the analysis, we used standard qualitative research methods using the basic procedures of grounded theory methodology and the constant comparative method [24, 25]. Using this methodology, the same two team members trained in qualitative research methods inductively analyzed the transcripts, generating theory from the data. The text was initially reviewed line-by-line and coded to characterize comments and passages. The transcripts were then re-evaluated to group codes into conceptual categories. Following coding of the initial set of transcripts, the entire research team met to discuss the findings and developed an initial coding scheme. The team then independently coded the next two transcripts, incorporated the new data into the initial conceptual categories, refined the existing categories to allow for a better ‘‘fit’’ of the new data, and added new categories as needed. After consensus for the refined themes was established, this process was continued with the same two members of the research team coding each of the remaining transcripts, followed by meetings to discuss coding discrepancies and make final coding decisions. The authors then completed final analyses, identifying overall themes and conceptual categories, and selecting the most reflective quotations. Following identification of the themes, as our final analytic step, we mapped the themes that emerged from the inductive coding process back to the core constructs of the PARiHS framework (evidence, context, facilitation) examining the strength or weakness of each to provide us with an understanding of the successes and challenges of the implementation. (See Rycroft-Malone, 2004 [23], Table 1 for a detailed description of the elements of the PARiHS framework and the distinction between high versus low levels of each sub-element).

**Table 1:** Characteristics of study participants (n = 50)

Characteristic	N	%
Sex		
Male	22	44
Female	28	56
Age		
<25	17	34
36–45	8	16
46–55	11	22
56–65	14	28
Years of work in this ED		
1 or less	8	16
2–5	16	32
6–10	8	16
11–20	9	18
>20	9	18

## Results

### Description of the study sample

The final sample of 50 participants included 19 physicians, 26 registered nurses, 3 HPAs, and 2 pharmacists. Table 1 describes basic demographic and practice characteristics of the study participants. The participants were predominately female, with half over age 45. More than half had worked in this ED at least 6 years. With the exception of the resident physicians, who by the nature of their role had worked in the ED for 4 years or less, length of service among the nurses and attending physicians ranged from 4 to 25 years.

### Overview of qualitative themes

We used the focus group and interview data to provide us with an in-depth understanding of the participants’ views of the policy implementation. Emerging from our analysis was a series of themes related to barriers and facilitators, as well as multiple suggestions for improvement that were grouped into thematic categories. Primary themes related to facilitators included: (A) the intervention being real-world driven with philosophical, clinician and leadership support; (B) the implementation of some early basic education and training efforts; (C) availability of resources (e.g., 24 h availability of staff); and (D) ability for patients to actually leave the ED with the naloxone kit in hand. Yet, relevant to the lack of uptake of the intervention, the barriers were multiple and fell into the following five more general categories: (A) protocol and policy-related barriers; (B) workflow and logistical barriers; (C) patient-related barriers; (D) barriers related to staff roles and responsibilities; and (E) education and training barriers.

### Facilitators

#### Philosophical stance of support for naloxone distribution

There was a strong sense of support for the intervention for a multitude of reasons. It was considered to be real-world driven and consistent with both the mission of the hospital as a major safety net provider, as well as the current community and political climates. As a safety net hospital, ED staff embraced distribution of naloxone kits as a practical tool closely aligned with the overall public health mission of prevention. Kit distribution was viewed as in sync with statewide and national awareness of addiction as a disease, the severity of the opioid overdose crisis, and the need for a multi-pronged approach to address the crisis. A member of the ED leadership team said the following:

I don’t think you’re going to find much philosophical resistance here… You know, I think for this department, the public health mission is who we are. So I think that there’s an overall attitude of it is great and exciting to take care of illnesses and shootings and overdoses. But it’s just as exciting to prevent the next one. So I think that clearly this is a receptive environment, I think on the whole. (Interview 3).

A second component of the philosophical support was that naloxone was clearly viewed as an evidence-based intervention in terms of its effectiveness. The philosophical stance of support was apparent at all levels, from hospital leadership and ED administration to the range of clinical and support staff. An illustrative quotation from a focus group described the general tenor of ED staff: *Why would you be against Narcan? I can’t even think of a rational argument….It’s like it’s something like condoms or…that’s like being against water…* (Focus Group 7).

#### Education and training foundational efforts

Despite the low uptake, some groundwork to support the implementation of the policy was initiated. Participants believed that, for the most part, there was general familiarity with policy. Personnel were notified via email and electronic communication of the policy by the physician champion of the policy within the ED, and were also provided with some basic one-on-one information. Communication techniques were tailored to the fast pace of the ED and included “catching” people when possible when they were working, although this was not done systematically. Resident physicians were familiar with the HPA model, but less aware of the specifics of how to prescribe naloxone rescue kits themselves or order them through the inpatient pharmacy. In addition to basic information given by the physician leading the effort, nurse educators, who have the primary responsibility for the conduct of training, made efforts to inform ED staff about the policy and spread the policy in multiple ways. They utilized electronic communication, conducted in-services, had pharmacy staff train staff nurses in use of the naloxone kit, put up posters, and obtained permission from the local department of public health to upload their videos to the nursing webpage.

#### Availability of resources

Certain resources were available in the ED to support naloxone kit distribution, ensuring this initiative was taken more seriously than other unfunded mandates. Specifically, the HPAs were trained and provided naloxone rescue kits via the state prevention pilot program. Physicians and nurses pointed to the crucial role of the HPA, including both having the time to distribute the naloxone kits, and critically, having the time and expertise to deliver the education components. Further, participants acknowledged two new resources becoming available at the time of policy implementation that support the effort and potentially improve uptake: a 24-h pharmacy within the ED and 24-h availability of social workers on a daily basis.

#### Ability to leave the ED with a naloxone kit

Along with the option to prescribe naloxone for patients to pick up at an outpatient pharmacy, this ED has naloxone available on-site to distribute to patients, allowing them the option to leave the hospital with the kit in hand. The HPA program naloxone kits are provided by the state’s public health agency and workers can hand these to patients (and interested family members or supportive others) at no cost. This option allows for simple and streamlined access to the actual kit and avoids the extra step, and potential stigma, of going to the pharmacy to pick up the kit with the prescription. Kits can also be distributed by the ED pharmacy during hours when the HPAs are unavailable. The policy was intended to allow for easy distribution by a range of providers via the hospital standing order and the ability for any member of the ED staff to theoretically obtain the naloxone kit and provide it to a patient. One provider described the importance of easy access:

If it’s not in their hand walking out the door, if they need it, they got too many other things on their plate to go worry about as opposed to go get it. And if they don’t need it, then it’s not a big problem. So unless you can hand it to people, I would bet that the follow through is low (Interview 2).

In addition, another facilitator was that the policy allowed for the immediate distribution of the kit to family members and caregivers who come to the ED with a patient, which one participant describe as potentially very welcome by and accessible to family members, as well as more accessible to individuals as risk for overdose:

I think in an ideal world, somebody would walk out with it…I mean, in some ways it’s a little counterintuitive because people are more than happy to wait in a line at CVS for their Percocet prescription. But if they happen to get that—*but they’re very less likely to*—*when they’re kind of in the midst of this kind of overdose haze to walk in. The family might do that, you know? A mother, brother, sister, father might do that. But I think a lot of other*–*you know, the person who’s abusing substances themselves or their immediate surrounding kind of peer group, I’m not sure.* (Interview 1).

### Barriers

#### Protocol and policy related barriers

There were multiple protocol and policy related barriers. First, the policy was felt to have been developed with insufficient input from frontline staff. The people working in the ED who do the actual work and implement the policy were not involved in its development. As one physician stated, *…the people who designed the policy…don’t work nightshifts…* (Interview 6) and thus would not understand the unique challenges of implementation. This was coupled with an under-developed implementation plan to accompany the policy. Additionally, the physician who signed the hospital standing order was not an ED provider and there was strong consensus that many individuals simply did not know who this person was; the lack of familiarity led to hesitancy to use the order.

Second, there is a major challenge in identifying who is the “right” patient to be offered/to receive the naloxone kit. While the policy includes patients who have overdosed and many categories of patients felt to be at risk for overdose, there is confusion and lack of consensus over who the appropriate kit recipient is. Multiple participants described these challenges:

I think it’s hard. I think that the person who overdoses is by far the–*you can’t miss that person, it’s a red sign. But the patient who you go through their history and they say, “Yes, I use IV drugs, I use IV heroin,” but they’re not there for the heroin issue, but they’re there for maybe something unrelated to it, like chest pain or acid reflux or whatever else brings them in, a sprained elbow, that sometimes you can think about, “Oh, their primary problem is the elbow issue. It’s not that they’re all using IV heroin, I should address that today.” Because that’s not what they came for and that’s not necessarily what I jump to as the primary reason why they came, either… The older folks on chronic narcotics fill up half the ER most of the time anyway and I think that that population is just not*—*at least it’s not in my mind as much…And I think also the people who come in on 20 different meds, et cetera…* (Focus Group 1).

The absence of any clear, objective criteria led to lack of consensus. There appeared to be some areas of general agreement. For example, one participant stated offering the kit …*if I see in their prior visits that they’ve been to the ED for heroin overdose five times already. (Focus Group 5)* Another person, who indicated rarely offering the kit, stated:

I just don’t think about it a lot, or a few times I’ve run into people saying they didn’t want it and I never went beyond that. However, the ones that I do think about and offer it to, it depends on what they came into the emergency department for. And usually if it’s anything related to IV drug use, whether it’s an overdose or an abscess from IV drug use, is when I think about it. **(**Focus Group 3).

A third participant stated *I have given it to people who’ve come in for opiate*-*related complaints as well. Or if I have seen that they’ve been here a lot for opiates, but not necessarily with this visit.* (Focus Group 3).

However, despite some clear specific consensus targets for naloxone rescue kits (people who have overdosed, people who use injection drugs with multiple visits), overall there was not consensus on who warranted receiving a rescue kit and who did not. While some hospital leaders believed that anyone prescribed an opioid should be offered naloxone, many providers strongly believed that this was not feasible, realistic or appropriate. As one individual stated,

Well, in a resourced world where you have somebody who could really do effective navigation… that was the discordance when I really looked at the policy… that just getting to an at risk population versus those who have evident and immediate risk of death, I think you got to– *in a world of limited resources, you have to decide where you’re going to devote your time and effort.* (Interview 6).

At the same time, there was concern that the policy was developed without thinking through where it was most applicable: for example, while interviewees believed that probably one of the most receptive groups are the parents of the younger people, the perception was that there was no involvement with or participation of the pediatric ED.

A fourth policy-related barrier was the uncertainty about what the ED parameters for naloxone kit distribution are. Despite the strong sense that this ED has a public health, preventive mission, there was a feeling that it is important to clearly define the appropriate role of the ED in these types of public health functions, wondering about the role of the ED to educate or just address immediate life-threatening issues. Some participants desired naloxone kit distribution to be similarly to the simplicity of routine distribution of condoms. Others likened it to providing a prescription for an epi pen following an allergic reaction. These ideas were raised multiple times and encapsulated in a focus group:

In urgent care, there is a…a basket of condoms, which is a public health concern and lots of things and people literally on their way out the door grab a handful….every once in a while a kid walks by and thinks it’s candy and his parents have to kind of take their hand away, which may bring some awkward conversations to the ride home. And I understand Narcan is different, it’s a drug there are different rules and all that sort of stuff. Can’t buy Narcan at the pharmacy, but you can buy condoms at a pharmacy, I get all that. But the concept is exactly the same. (Focus Group 7).

Another provider said the following:

If I were to model it on treatment of allergic reactions, we identify people routinely when they have a screening drug history that they have anaphylaxis to penicillin. Theoretically, they should have a med alert bracelet, they should carry an epi pen, we should write a prescription for them because they are at risk of having–*or they have a peanut allergy. Is that really the role of the emergency department?* (Interview 6).

#### Work-flow and logistical barriers

Workflow and logistical barriers were myriad and many were attributed to poorly thought-through implementation issues in the development of the policy. First, the concept of a ***standing verbal order*** was a foreign and utterly unfamiliar work flow process, particularly among the nursing staff, who stated this was the only policy structured this way and was totally inconsistent with current practice. As explained by nurses in one focus group, there is no mechanism for standing verbal orders *…because we’re told we’re not allowed to take verbal orders…* and *We’re only allowed to take verbal orders in life*-*and death emergencies….* (Focus Group 3); Second, while the hospital standing order theoretically meant that no written order or prescription was required to obtain the naloxone kit from the ED pharmacy, in practice, paper documentation was required by the pharmacy to dispense the medication, while all other documentation to dispense medications was done electronically.

Another logistical and workflow issue related to the electronic medical record (EMR). At the time of policy implementation, the institution was introducing a new electronic medical record system. Staff needed to become familiar with the new system and the system initially did not include naloxone either as an option for ordering for a discharge, making staff unable to easily order it. Moreover, asking about overdose history/risk was not included as a part of the standard set of questions in the standard workflow/templates within the EMR.

Beyond the EMR, there were multiple barriers to ***physically accessing the naloxone kit and getting it to the patient.*** The HPAs hold and administer the free kits provided by the public health department. For non-HPA staff to provide kits to patients from the hospital pharmacy, the process for ordering was not simple or clear. Those who tried to access the actual kit indicated that obtaining it was extremely challenging and included multiple and unclear steps. Pharmacists indicated that they could be available to access the kit, deliver it and potentially even educate patients on its use. However, not being directly involved in patient care, there was not a clear and consistent trigger to notify them that a patient needed a kit.

Another key barrier identified was ***lack of clarity/consensus on the best time to distribute naloxone***. Both physicians and nurses expressed that discharge may not be the optimal time to offer it, with a common sentiment being

…I think the hard part is we wait for the end when they’re ready to go and half these patients, we all know, they’re on their way out, you know. They’re not going to wait for their discharge paperwork, never mind the…Narcan… (Focus Group 7).

The following exchange between two nurses in a focus group illustrates the logistical challenges that staff faced, even when very motivated to provide a kit:

***Participant C***: I gave one to a father one night who was here with his kid. And kind of at his wits’ end. There was no detox to get him into. Father was kind of “what am I supposed to do?” And the physicians and I talked about it, and we got one. With a lot of difficulty… Got it from the pharmacy. And I mean, I had to pull up the emails that this was going to be able to be available to give it to the pharmacist in order to get it. It was the only ….

***Participant D***: It was really difficult.

***Participant C***: It was very difficult. The father was very appreciative. It was the only one I’ve done.

***Participant D:*** It’s like we got the emails and nobody in the pharmacy got the emails. We all knew it was available and they wouldn’t give it out. It was horrible, trying to get it. Way more work than it was worth. (Focus Group 3).

#### Staff perception of patient-related barriers

Staff perception of patient related barriers to naloxone kit distribution were frequently raised beyond deciding who the right patient is to be offered the kit. There was a hesitation to vocalize what could be interpreted as a pejorative description of *the profile of patients,* particularly those who are admitted with an overdose and other drug-related issues. It was stated by most staff that the patients can be very challenging. Despite the medical understanding of addiction and the fact that patients are experiencing withdrawal from the naloxone—meaning their brain and body need opioids not to feel “dope sick”–their impatience and behavior of wanting to leave the ED should be expected. And yet, comments included that the patients do not care that they overdosed; they tend to be an impulsive group that does not want to wait, they may be anxious and want to leave once the overdose has been reversed. The following interchange occurred between two participants:

***Participant A:*** …it’s my own opinion…but from when I talk to people…say they’re a very difficult patient population to care for. They’re not the nicest people. A lot of times they are, as Participant B said earlier, anxious or they want to leave, they want to go smoke. They have all these other kind of counter habits that go along with it. And sometimes the nurses say they would approach them to do some of the teaching and the patient itself would say, “Oh, no, I’m not interested in that, get that away from me, I have an allergy to that.”

***Participant B:*** Yeah…going along those lines, they, a lot of them have come back to life after an OD in the ER and they’re ready to go. They don’t want to even wait for the safe amount of time…Or they’ve had the experience of having it administered and they don’t want any part of anyone knowing about it. (Focus Group 5).

Another participant stated that some of the *patients …did not want their ‘high’ to have been reversed by Narcan and so are not interested in getting some to go.*(Focus Group 6).

Further, many patients who are substance involved have co-occurring life stressors and struggles including housing, vocational and economic instability, and are often the target of societal discrimination and scorn. Given these challenges faced by many of the patients, there was a feeling that the option of providing a prescription rather than an actual kit was a barrier in that patients are unlikely to fill it due to inconvenience, stigma, and possibility of incurring an insurance a co-payment. In addition, patients who have overdosed tend to be alone in the ED. Many staff interviewed note that the target recipient of kits should be family and friends of the patients rather than the patients themselves and suggested it was perhaps more important to think about alternative mechanisms beyond the ED visit to reach out to these partners and family: *…It’s reaching their partners. I think it’s reaching their family…..because the patient isn’t interested.* (Focus Group 6).

Finally, despite general support for naloxone kit distribution in the ED, a vocal minority of ED staff across disciplines raised important questions regarding what they see as a potentially unspoken, but important barrier to kit distribution. This relates to their perception of ***who is “worthy”*** to receive naloxone. Some wanted patients to demonstrate motivation in order to be offered the kit. They expected that a patient “pull oneself up by the bootstraps” rather than be enabled:

*But for me, the patient has to show some motivation. I mean, I’ll offer it. If they’re interested, they’re going to get one. If they really blow me off, I’m not going to stick one in their backpack. They need to show some motivation if they want it. I do have to be given that.* (Focus Group 3).

One provider talked about some of the challenges staff face: *I think the reality is you can change people’s behavior in certain ways, you can’t always change their beliefs.* (Interview 2). Another person gave the opinion that all of the staff likely had feelings about who is worthy that are tied up in beliefs and feelings about whether addiction is self-inflicted or a disease:

I think that partly people are part of the culture that they’ve grown up in and people that use drugs, unless it’s somebody you know or a family member, it becomes very stigmatizing. I think sometimes the patients can be very, very challenging to deal with and so that also sort of weighs into it, I think. That people still don’t understand. You came in with endocarditis once before and almost died, and you’re still shooting and you’re back here again with the same problem. What’s up with that? Yeah, I think there’s still a sense of– there’s the deserving and the undeserving ill and this is self-inflicted and not disease. I don’t think people in healthcare are immune to that…. I think people who work in healthcare, A, come from different perspectives, but also they’re human beings last time I checked. So, we have our own biases. We grew up in a culture where some behaviors are acceptable and not. (Interview 6).

#### Staff roles and responsibilities

It was almost unanimous that no one knew what staff member or members were responsible for naloxone kit distribution, other than the HPAs when they were available. While the concept behind the hospital standing order is that every staff member, at all levels, could provide a kit when appropriate, that was not considered realistic. Repeatedly stated were comments related to kit distribution not being in anyone’s job description or not being anyone’s responsibility.

When asked specifically whose job they believed it was, there was little agreement, either by discipline or by level. There was strong belief among staff with clinical responsibilities that they are too busy and have too many competing demands, and they have conflicting ideas about who can/should do it: *I have no doubt if you ask nurses who should do it, it’ll be physicians. And if you ask physicians who will do it, it will be nurses….*(Focus Group 7). Physicians said that their understanding was that nurses would be doing this after hours when the HPAs were unavailable: *…it makes sense to me in that the nurses are the end point of discharge, particularly off hours, where it’s part of their role and responsibility, I think, to make sure the patients have a discharge plan….* (Interview 6).

On the other hand, nurses strongly believed that dispensing medication is not part of their nursing role, inconsistent with current practice, and raises questions about legality and the impact on their nursing licenses. One nurse said *No other medication is the nurses’ responsibility to give without an order from a physician,* while another said*, No. No, we would not routinely take orders that aren’t directly written by the on*-*duty physician.* (Focus Group 3).

At the same time, while acknowledging the high level of clinical responsibilities of residents, some attending physicians believed this was the residents’ responsibility, stating *there are always residents.* (Interview 6) However, those in charge of the residency believed strongly that this was not an appropriate role for the residents. Though many participants believed that kit distribution and patient education could be a pharmacy or social work role, the pharmacists suggested it should be the role of a person with clinical responsibility, and specifically residents. The HPAs believe it is only a small part of their job, despite other staff perceptions to the contrary. Overall, while the concept of kit distribution being a responsibility of all staff was, in many ways, considered a good model, there were diverse opinions about this and it was acknowledged that it could be challenging. One resident aptly described the challenges of this being a somewhat universal function in the ED environment:

I think that gets at a bigger issue we face as a busy emergency department with a lot of people having times when they have to step out of what they typically– or what you think your defined role might be, to actually make stuff happen. So I think that on the one hand, it could make it be that everybody says it’s not my job. And there’s certainly frustration around a lot of things where that happens. But at the same time, at times if there’s only one person who’s responsible for doing it, they have a lot of other responsibilities, too, and it’ll make things slow down or not happen…So I think there’s probably not a perfect answer, but for most everything else in our department, it falls under everybody sort of has to do everything depending on the circumstances. And I think this would just sort of go along with that as our practice and culture here. (Focus Group 1).

It was also noted that the mechanism and responsibility for kit distribution vary depending on the shift and ED staff work rotating shifts. This was viewed as confusing to many participants. For example HPAs are not available during the overnight hours (3rd shift). Yet, due to reliance on the HPAs on other shifts, staff working on the 3rd shift may not actually know the steps to take that result in a patient being discharged from the ED with a kit in hand. Another example involves the differing role of pharmacy by shift and the hours of operation of the various hospital based pharmacies. Due to regulatory issues, the hospital based pharmacy can only directly dispense the medication in the ED during off hours when the outpatient pharmacy is closed. As a result, each time of day there is a different process.

#### Education and training gaps

The final barrier identified was a set of training and education gaps for staff and patients. Staff members need two types of training: training in the policy and its implementation; and training on how to use the naloxone kits. Staff consistently spoke about not knowing how to use the kits themselves and thus, how to best teach patients how to use the kits. Many indicated that their only experience addressing substance use needs beyond diagnosis was referring to the HPA program.

Staff also uniformly felt that patients and families need training about what naloxone is, how it works, and how to use the kits. There was a strong belief that the kit is not simple to use and educating patients is a big time commitment. Thus they believed it was important to determine how to *incorporate patient training into the work flow*, as time pressures and other responsibilities make it difficult for staff to do actual patient training.

## Discussion

Results from the focus groups and interviews revealed participants’ insights about the influences and consequences of the new policy. The results help to illustrate how the actual implementation of a new policy or health care delivery intervention is largely driven by factors beyond acceptance of the policy or intervention. Despite strong philosophical support at all levels and devotion of select resources, the implementation of the new naloxone distribution policy in action was challenging, with low uptake. Examining this using the PARiHS framework constructs of evidence, context and facilitation provides important insight into the results and perceptions, and supports improvements in policy implementation.

### Evidence

In the PARiHS framework the construct of evidence includes research evidence, clinical experience, patient experience and local data or information [23, 26]. In terms of research evidence, responses were unequivocal regarding naloxone itself; no one questioned its safety and efficacy. Notably, the ED staff did not identify some of the other concerns raised in the literature regarding the safety and effectiveness of nasal naloxone in terms of risks, such as: side effects of home administration; not seeking care; or increasing the risks of using more opioids to overcome the effects of naloxone [27–29]. In addition, while physician participants did not raise concern about the medicolegal risks to prescribers found in other studies [30], this was a primary concern among nursing staff in our study, who do not have authority to prescribe medications.

On the other hand, the research evidence about the effectiveness of providing naloxone kits as prevention in the ED setting was viewed as lacking. Specifically noted was the absence of any clear, objective criteria or clinical guidelines defining what it means to be at risk for overdose leading to lack of consensus for identifying who to offer the kit to. Because ED-based naloxone kit delivery is not a standard, evidence-based practice, it will be important to concretely identify–based on evidence that does exist from other settings–who is at risk for overdose death to assist the ED in identifying a clear population target [9, 30, 31].

Clinical and patient experience, key components of evidence according to PARiHS, created substantial barriers to implementation. ED patients presenting with an overdose or other drug-related issues were viewed as extremely challenging, impulsive and potentially unwilling to accept or wait for the kit to be delivered to them. Clinical experience showed providers that, given the challenges faced by many of the patients, simply providing a prescription was insufficient. Moreover, clinical experience led the group to consider that a better model of delivery would be to offer the naloxone to family and/or friends of the patients rather than the patients themselves; however, many patients are alone in the ED.

Patient experience was a barrier, according to staff. Withdrawal symptoms after overdoses had been reversed by naloxone made patients unlikely to want to experience naloxone again. Although naloxone rescue is lifesaving, it is not immediately appreciated by the overdose survivor, who is often experiencing very uncomfortable withdrawal symptoms. The ED has little to offer an overdose patient who has just been administered naloxone and is now in opioid withdrawal other than observation to ensure that the overdose does not recur. When detox programs are available, patients typically wait several hours with progressively worsening withdrawal symptoms. Immediate access to opioid agonist treatment, for which there is strong evidence [32], is not routinely accessible through the ED. Opioid addiction conditions people to do all that they can to avoid withdrawal. When they experience withdrawal, their focus is on relieving their symptoms by using opioids. It follows that a patient in withdrawal after an overdose is not in the best state to receive overdose prevention education and training to administer naloxone. Although patients with emergencies are often accompanied by caregivers, culture and processes to offer emergency response training and prevention education to caregivers within EDs are not well developed. Thus, focusing on caregiver training for the overdose survivor is likely a strategy that may be good for the patient and that ED staff are open to with the right level of training and resources.

Finally, there was the sense that local opinion or data were not reflected in policy development, specifically: who should actually be responsible for implementation/who had the time to offer naloxone; optimal location for implementation; how to physically access the kits; and documentation processes. These details are especially important, as studies have shown that implementation of new interventions in hospital settings is extremely challenging and fraught with barriers, even if the new interventions are widely accepted evidence-based practices [33, 34]. Thus, the implementation of a practice not known to be evidence-based to a particularly challenging patient population is likely to be logarithmically more difficult.

### Context

Several contextual factors related to PARiHS sub-elements: culture, leadership, and evaluation [23] were high. The institutional culture was a key factor in the concept of the policy being strongly embraced. The intervention was considered to be real-world driven and consistent with the mission of the hospital as a major safety net provider and one potential response to the current local and national opioid overdose death crisis. At the same time, the supportive sentiment was tempered by the desire to determine if this stance was an appropriate one for the ED. Part of culture is provision and allocation of resources; resources were provided to support implementation, although there was not widespread or full awareness of how to utilize those resources. Although naloxone kits were available, often free of charge, there were numerous workflow and logistical barriers faced by the staff that had to implement the policy or obtain the kits that impeded implementation. Having a paper order in an electronic system is one such example.

Our findings are consistent with the literature on prescriber motivation and interest in naloxone rescue kit distribution which identifies reticence around role clarity and appropriate patient selection [29, 35]. This is best illustrated by the nursing staff’s indication that the standing verbal order was inconsistent with current practice, their concern about their medicolegal risks in ordering medications, and the near-universal confusion across all staff groups regarding whose job this was. The need for an extremely high degree of role clarity is particularly important for a task that involves a different process utilizing unequally available resources on each of the 3 shifts, particularly when staff may rotate among the different shifts. Role clarity has been found to be critical to job performance [36–38]; if roles for individuals are clarified, implementation may be improved, as studies have shown that EDs can effectively deliver public health-related safety interventions and messages [39, 40].

### Facilitation

Facilitation includes the role played by the leader, as well as the skills and attributes possessed by that leader [23] in enabling the implementation. In this intervention, facilitation was internal and was not led by a clearly identified clinician champion, although the ED-based leader of the initiative served as a de facto champion. There were not sufficient resources or time to support systematic ongoing training in a structured manner. Thus, training was episodic and inconsistent, with the primary method of communication and training being via email, posters, or intermittently by clinicians who supported the policy, but were not the clearly identified owners. As a result, many staff indicated little to no awareness of the policy, while resident physicians who received some of their training from the clinician champion did indicate basic familiarity with it and recalled being taught about it in their training, although they were frequently unaware of the specifics. This is consistent with the literature indicating that few physicians are even aware of ED-based naloxone prescribing as an intervention to prevent overdose [27].

It is also important to widely engage staff. Studies have shown the importance of using mid-level managers, rather than just clinicians and leadership to implement innovations in health care [41] and this team did try to actively engage nurse managers and nurse educators, a positive step. Yet, that engagement was not ongoing, systematic, or consistent. In addition, a facilitation barrier was that—out of necessity-the policy was implemented quickly, without sufficient time for a thorough and consistent implementation plan or consideration of other simultaneous and competing initiatives. For example, policy implementation occurred while the hospital was introducing a new EMR that did not include the option of naloxone kits as a discharge order. Additionally, another resource was provided relatively early in the implementation period via by increasing ED-based social work coverage to around the clock, with the social workers potentially available to train patients and distribute kits; however, this was hastily introduced to the rest of the ED staff without clarity regarding their role related to this process. Thus, rather than supporting the effort, it created confusion.

### Suggestions for improvement

Despite the challenges of implementation, people offered multiple suggestions to improve the implementation to ensure that naloxone is offered to individuals who could benefit. These included: (1) offering a clear definition of what it means to be at risk of overdose; (2) narrowing the target population in the initial stages of implementation to those at highest risk, such as those who have overdosed; (3) streamlining the process to mesh with the workflow, including the use of an EMR-based process that could include a template or an “opt out” option; (4) clear and specific identification of staff roles and assignment of responsibilities; (5) identification of site champions; (6) instituting consistent training for all ED staff; (7) developing a training program for caregivers and families; (8) creating a method to ensure integration of patient and family training into the ED workflow; and (9) conduct regular audit and feedback to ED staff. Due, in part, to the preliminary results and the suggestions from participants, there have been some modifications. Modifications include integrating naloxone as a discharge medication into the EMR, increasing ED pharmacy availability to 24 h per day, and training house officers on use of naloxone as part of their basic life support training and on the policy. Early findings following introduction of these changes showed a modest increase in naloxone distribution in the ED, with internal data indicating 39 naloxone kits distributed in October 2015.

### Limitations

This study has some limitations. First, participants were recruited from a safety net institution in Massachusetts that had a high level of access to naloxone kits, and therefore, their views might not generalize to ED providers in other geographic locations. Second, although we conducted broad recruitment, participants self-selected to participate and those who agreed may have agreed because they had particularly strong views about the overdose epidemic, drug use and/or the intervention. In addition, as in any focus group study, although participants were assured of confidentiality and their responses appeared to be very candid, including a range of negative comments and concerns about the program and suggestions for improvement, we cannot exclude the possibility of biased or socially desirable responses. However, our goal was to examine the process of implementation of a policy, which may be highly generalizable and our findings have important implications for clinical practice.

## Conclusion

The problem of opioid overdose is at crisis levels and rising. Naloxone can reverse overdose and needs to be widely available. Multi-faceted, innovative solutions are needed to ensure that those who are most at risk for overdose have access. Thus, interventions such as the one proposed in this safety net ED are crucial. Yet, the uptake of this policy was limited. While the true potential of widespread access to naloxone rescue kits is unknown, understanding the experience of this safety net hospital is important to improve uptake in this setting and to offer possible solutions for other EDs. Use of the PARiHS framework throughout the study design and analysis, a strength of this study, allowed us to recognize key evidence, contextual and facilitation barriers to the successful implementation of the policy to identify areas for intervention and improvement. In particular, we recommend the identification of a limited and relatively focused target population with a high degree of risk to begin policy implementation. Use of diagnostic and-mechanism-of-injury codes (*International Classification of Diseases, Ninth Revision, Clinical Modification* [ICD-9-CM] codes) is one potential strategy for defining and identifying patients most at risk for overdose [42]. We recommend the development of the capacity and training to engage caregivers of overdose survivors in overdose prevention and response during an ED visit. Once successful with these targeted groups there is potential to broaden the scope. In addition, it is important to recognize that staff perceive substantial patient-related barriers, whether or not these barriers exist in reality and to address those perceptions through staff education. The signatory of the hospital standing order should be someone who works in the ED and is a recognized clinical leader. Thus the signatory can internally promote the standing order and be accountable to staff that have concerns. Furthermore, one clinical champion is unlikely to be sufficient. At least one clinical champion from each “role” in the ED should be identified and supported to promote the implementation of the overdose prevention policy within their respective roles and convey the opportunities and challenges to colleagues representing other roles. Finally, although the intent of the policy was that distribution of naloxone be a universal function, which was the reason for the standing order, it was apparent from interviews across all staff that this is not viable and that clarification of responsibility is vital. Clearly, the staff working are very invested in and supportive of finding methods to address opioid overdose, given its impact in this ED and nationwide, and would like to find methods to enhance the intervention; appropriately modifying the implementation process—paying attention to what was learned from this experience in terms of key components of evidence, context and facilitation—is likely to lead to improvements and can serve as lessons for ED and other busy safety net environments.

## Additional files

**Additional file 1.** ICD-9 codes for denominator.

**Additional file 2.** Intranasal naloxone kit discharge order protocol.

**Additional file 3.** Understanding Barriers and Facilitators to Implementing Routine Emergency Department-Based Overdose Education and Naloxone Kit Distribution [OEND] for Patients at Risk of Opioid Overdose.

## Abbreviations

ED: emergency department
PARiHS: promoting action on research implementation in health services
HPA: health promotion advocate
EMR: electronic medical record
