# Supplementary material for: Why is it so hard to implement change? A qualitative examination of barriers and facilitators to distribution of naloxone for overdose prevention in a safety net environment
Source: BMC Res Notes. 2016 Oct 18;9:465. doi: 10.1186/s13104-016-2268-z (PMC5070095; doi:10.1186/s13104-016-2268-z)
Supplement: Supplementary file 1 — Additional file 1. ICD-9 codes for denominator. [file 13104_2016_2268_MOESM1_ESM.docx]

**Additional File 1 - ICD-9 codes for denominator**

**1. Opioid Overdose**

965.00   Poisoning-Opium NOS   Poisoning by Opium (Alkaloid) NOS

965.01   Poisoning-Heroin             Poisoning by Heroin

965.02   Poisoning-Methadone  Poisoning by Methadone

965.09   Poisoning-Opiates NEC  Poisoning by Opiate/Related Narcotic NEC

970.1     Poison-Opiate Antagonist            Poisoning by Opiate Antagonist

E85.00   Acc Poison-Heroin           Accidental Poisoning by Heroin

E85.01   Acc Poison-Methadone Accidental Poisoning by Methadone

E85.02   Acc Poison-Opiates NEC                Accidental Poisoning by Opiate/Narcotic NEC

E93.50   Adv Eff Heroin   Adverse Effect of Heroin

E93.51   Adv Eff Methadone        Adverse Effect of Methadone

E93.52   Adv Eff Opiates Adverse Effect of Opiate/Related Narcotic

E94.01   Adv Eff Opiat Antagonist              Adverse Effect of Opiate Antagonist

**2. At risk for opioid dependence/use**

304.00   Opioid Dependence-Unspec      Opioid Type Drug Dependence NOS

304.01   Opioid Dependence-Contin        Opioid Type Drug Dependence Continuous

304.02   Opioid Dependence-Episod        Opioid Type Drug Dependence Episodic

304.70   Opioid/Other Dep-Unspec          Opioid Combination Dependence NOS

304.71   Opioid/Other Dep-Contin            Opioid Combination Depend Continuous

304.72   Opioid/Other Dep-Episod            Opioid Combination Depend Episodic

305.50   Opioid Abuse-Unspec    Opioid Abuse NOS

305.51   Opioid Abuse-Continuous            Opioid Abuse Continuous

305.52   Opioid Abuse-Episodic   Opioid Abuse Episodic
