# Supplementary material for: Why is it so hard to implement change? A qualitative examination of barriers and facilitators to distribution of naloxone for overdose prevention in a safety net environment
Source: BMC Res Notes. 2016 Oct 18;9:465. doi: 10.1186/s13104-016-2268-z (PMC5070095; doi:10.1186/s13104-016-2268-z)
Supplement: Supplementary file 2 — Additional file 2. Intranasal naloxone kit discharge order protocol. [file 13104_2016_2268_MOESM2_ESM.pdf]

Policy and Procedure Manual

**Policy #:**	13.09.45
**Issued:**	Sept. 2013
**Reviewed/ Revised:**	
**Section:**	Pharmacy

Intranasal Naloxone Kit Discharge Order Protocol

Purpose:

To establish a Standing Discharge Order Protocol and dispensing procedure for Nasal Naloxone Kit Discharge Prescriptions in the BMC Emergency Department.

Policy Statement:

This protocol allows for Nasal Naloxone Kits to be ordered by licensed personnel for patients *at risk for opioid overdose* who are being discharged from the BMC Emergency Department. Under the protocol, BMC Inpatient Pharmacy is granted authority to dispense Nasal Naloxone Kits as a discharge prescription when the BMC Outpatient Pharmacies are closed. BMC waives the payment for these prescriptions.

Application:

All Pharmacy and Emergency Department (ED) staff

Exceptions:

None

Procedure:

Background

- The Massachusetts Department of Public Health (DPH) approves community programs to provide overdose education and naloxone distribution services and train potential witnesses to an overdose in accordance with their guidelines.
- The Project Assert program (sponsored by the Boston Public Health Commission), is an example of a community-based program. When a Project Assert team member is on-duty in the ED, patients are enrolled in the program and provided with Nasal Naloxone Kits obtained from the Project Assert supply.
- Intranasal naloxone rescue kits can be prescribed via a regular outpatient prescription by any licensed prescriber to a patient who is at risk for an overdose regardless of the provider's or patient's enrollment in a DPH program.
    - Prescriptions may be sent to BMC's outpatient pharmacies for filling during normal business hours.

- The establishment of this Protocol augments the services provided in community-based pilot programs for patients admitted to the ED at BMC, but is not a part of Project Assert or any other DPH approved pilot program.

Protocol

1. When Project Assert is unavailable and the outpatient pharmacies are closed, a nurse or physician may fax a Discharge Prescription for Nasal Naloxone Kit Standing Order to the inpatient pharmacy for a patient who is at risk for opioid overdose.
    - The Discharge Prescription form will be placed in the patient's medical record.
    - Under this protocol, prescriptions are considered to be signed by Alexander Walley, MD (the requirement for an actual signature is waived).
2. At risk patients in the following groups may be prescribed an intranasal naloxone rescue kit:
    - Received emergency medical care involving opioid intoxication or poisoning
    - Suspected history of substance abuse or nonmedical opioid use
    - Prescribed methadone or buprenorphine
    - Receiving an opioid prescription for pain:
        - higher-dose (>50 mg morphine equivalent/day)
        - rotated from one opioid to another because of possible incomplete cross tolerance
        - Smoking, COPD, emphysema, asthma, sleep apnea, respiratory infection, or other respiratory illness or potential obstruction.
        - Renal dysfunction, hepatic disease, cardiac illness, HIV/AIDS
        - Known or suspected concurrent alcohol use
        - Concurrent benzodiazepine or other sedative prescription
        - Concurrent antidepressant prescription
    - Patients who may have difficulty accessing emergency medical services (distance, remoteness)
    - Voluntary request from patient or caregiver
3. The inpatient pharmacy will prepare a Nasal Naloxone Kit containing:
    - Two Naloxone 1mg/ml 2ml luer-lock prefilled syringes
    - Two mucosal atomization devices (MAD300)
    - Risk factor information and assembly directions
        - DPH Opioid Overdose and Prevention Programs Information Sheets
        - Step-by-step instructions for administration of nasal naloxone
        - "Get the SKOOP" information pamphlet from DPH
4. Each kit shall be labeled in accordance with MA Board of Pharmacy standards, including the phone number for the BMC Outpatient Pharmacy at Shapiro.
5. The kit will be sent via pneumatic tube to the ED for the nurse to provide to the patient.

6. Each kit contains educational material for the patient. The nurse or prescriber should educate patients about the risks of opioid-related overdose, how to reduce them and the importance of seeking help by calling 911 in the setting of a suspected overdose.

Responsibility:

Emergency Medicine Staff, Nursing and Medical-Dental Staff, Pharmacy

Forms:

Order Form:

- BMC ED Discharge Prescription for Nasal Naloxone Kit

Pharmacy Label

- Print from Centricity using the Free Format label

Patient Education Materials:

- Naloxone_Instruction-Sheet_110112.pdf also see http://www.prescribetoprevent.org/wp-content/uploads/2012/11/naloxone-one-pager-in-nov-2012.pdf
- DPH-Info_Opioid-Overdose-Prevention-Programs_020413.pdf also see http://www.mass.gov/eohhs/docs/dph/substance-abuse/naloxone-info.pdf
- "Get the SKOOP" pamphlets are available from Massachusetts DPH 617-624-5111.

Related Policies:

Section:
Policy No.:
Title:

Initiated by: Pharmacy

Contributing Departments:

1. Medicine
2. Emergency Department
