# Supplementary material for: Why is it so hard to implement change? A qualitative examination of barriers and facilitators to distribution of naloxone for overdose prevention in a safety net environment
Source: BMC Res Notes. 2016 Oct 18;9:465. doi: 10.1186/s13104-016-2268-z (PMC5070095; doi:10.1186/s13104-016-2268-z)
Supplement: Supplementary file 3 — Additional file 3. Understanding Barriers and Facilitators to Implementing Routine Emergency Department-Based Overdose Education and Naloxone Kit Distribution [OEND] for Patients at Risk of Opioid Overdose. [file 13104_2016_2268_MOESM3_ESM.docx]

***Understanding Barriers and Facilitators to Implementing Routine Emergency Department-Based Overdose Education and Naloxone Kit Distribution [OEND] for Patients at Risk of Opioid Overdose***

***Brief introduction:***

- *Welcome/thank you/project description/focus group overview/consent and overview of*

*Overdose Education and Naloxone Kit Distribution [OEND] protocol*

**Interview/Focus Group Discussion Guide:**

The **OEND** Policy:

- Discussion of new **OEND** Protocol/Policy; what you had previously heard about the policy
- Please detail the parts of the policy you see working /not working (and reasons why). Do you feel this policy is feasible/not feasible to implement? Why/why not?
- What patient population do you see the policy focusing on (Those who have overdosed? Those at risk to overdose?)
- What do you feel are the challenges to implementing this policy? (BMC/your colleagues/work environment/logistics/etc.)
- What solutions can you suggest that might help overcome some of the challenges to implementing this policy?
- Any further suggestions or recommendations on how this implementation process might be improved?
- What do you think your colleagues think about this policy? Is there administrative/leadership support for the policy? (If yes, has this been helpful? If no, what administrative or leadership support might help in implementing this policy?)

Roles of various ED providers:

- In what ways to you see preventing future overdoses part of your role as an emergency medicine provider? If so, how do you see it as part of your role? (Probe as appropriate of role—RN vs MD, etc.).
- Who do you think should be responsible for ordering this kit? (If nurses: Although there is a standing order, do you think it is outside the scope of nursing practice to order this at discharge?)
- Have you ever given a naloxone rescue kit to anyone (patient, family member, significant other) at discharge? (If nursing, did the doctor order it?)
- Can you describe a scenario where you decided to give/ to not give a kit? What factors influenced or shaped your decision? What patients do you think could benefit from this kit?

(What makes someone an appropriate candidate to receive the kit?)

Project ASSERT:

Are you aware Project ASSERT’s OEND program? Have you referred patients to Project ASSERT? (why/why not). If not, would you be willing to refer patients to Project ASSERT?

Harm Reduction (concepts of):

- Please describe your exposure to/ experience with patients who use drugs and alcohol.
- Please describe your exposure to/ experience with patients who have overdosed (in ED/Other places?)
- What do you know about naloxone? How have you seen naloxone used during an overdose? (Issues/thoughts related to indications, side effects, administration of?)

Training & education:

- Please describe any training you have received in the ED around the new BMC OEND policy.
- Other than a naloxone rescue kit, what education or other interventions do you think are useful to reduce the risk of a person overdosing or dying from an overdose? If you do not find naloxone rescue kits to be a useful tool, why not?
- What overdose prevention tools would you prefer to use? How can naloxone rescue kits become a useful tool for your ED practice?

In general what types of trainings are most helpful /meaningful for you (video/article/meeting/presentation/etc.)?

Is there anything you want to discuss that has not already been discussed?
